# Supplementary material for: Thalamocortical orchestration of human theory of mind
Source: bioRxiv. 2026 Jul 30:2026.07.28.741302. Preprint. [Version 1] doi: 10.64898/2026.07.28.741302 (PMC13618414; doi:10.64898/2026.07.28.741302)
Supplement: Supplementary file 1 [file NIHPP2026.07.28.741302v1-supplement-1.pdf]

bioRxiv preprint doi: https://doi.org/10.64898/2026.07.28.741302; this version posted July 31, 2026. The copyright holder for this preprint (which was not certified by peer review) is the author/funder, who has granted bioRxiv a license to display the preprint in perpetuity. It is made available under a CC-BY 4.0 International license.

Supplementary

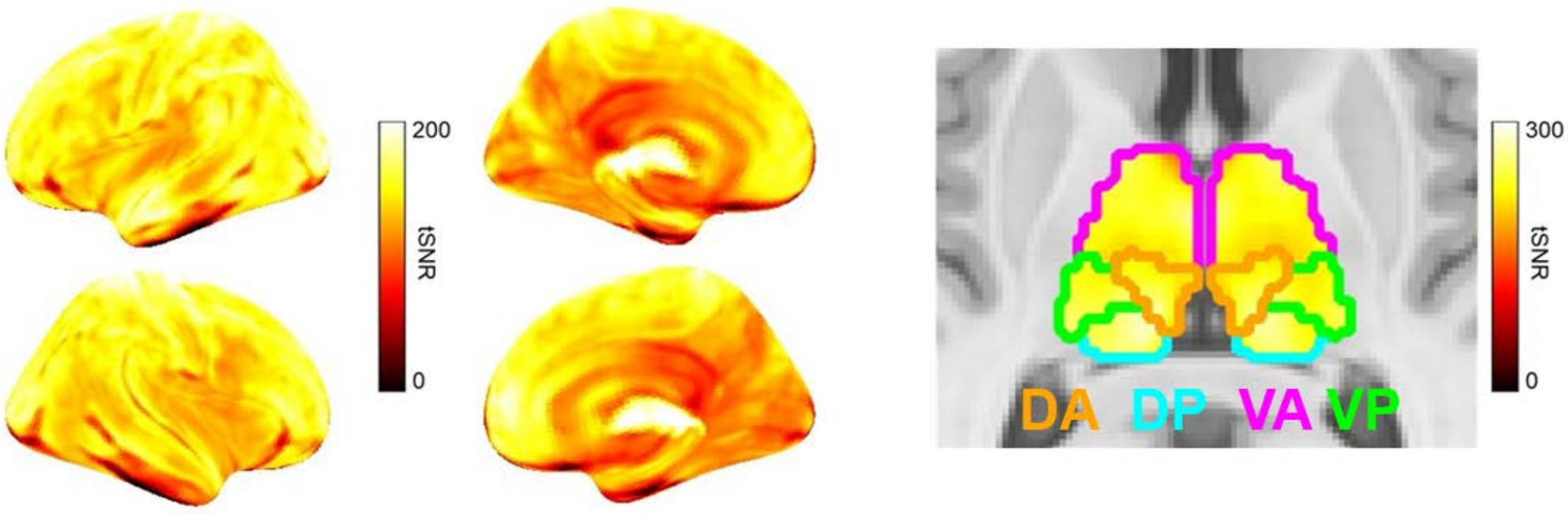

Figure S1. Temporal signal-to-noise ratio (tSNR) maps of the 7T multi-echo fMRI data. Group-averaged tSNR maps demonstrate robust signal quality across both cortical and subcortical regions.

bioRxiv preprint doi: https://doi.org/10.64898/2026.07.28.741302; this version posted July 31, 2026. The copyright holder for this preprint (which was not certified by peer review) is the author/funder, who has granted bioRxiv a license to display the preprint in perpetuity. It is made available under a CC-BY 4.0 International license.

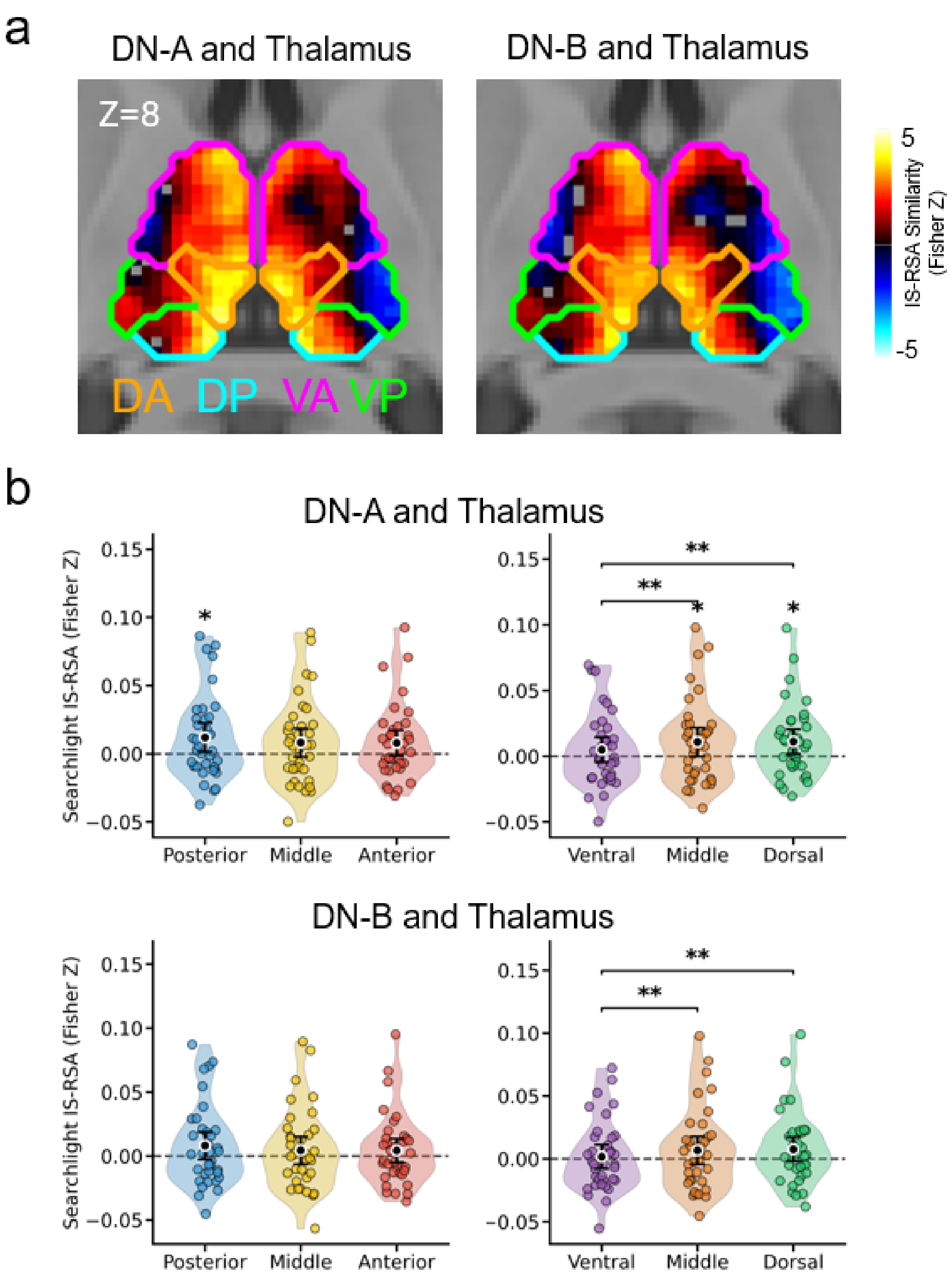

Figure S2. Sparse-sampling IS-RSA analysis. (a) Spatial topography of thalamocortical representational coupling revealed by searchlight sparse-sampling IS-RSA using a leave-one-out (LOO) framework. (b) Spatial binning analysis of the searchlight sparse-sampling IS-RSA along the anterior-posterior and dorsal-ventral axes. Each dot represents an individual subject, and error bars represent 95% confidence intervals. Pairwise comparisons among the three spatial bins were evaluated using two-tailed paired t-tests, while single-sample tests against zero (including the regional bin-specific tests and all ROI-level inferences) followed a directional one-tailed approach. * $p < 0.05$, ** $p < 0.01$ (FDR-corrected).

bioRxiv preprint doi: https://doi.org/10.64898/2026.07.28.741302; this version posted July 31, 2026. The copyright holder for this preprint (which was not certified by peer review) is the author/funder, who has granted bioRxiv a license to display the preprint in perpetuity. It is made available under a CC-BY 4.0 International license.

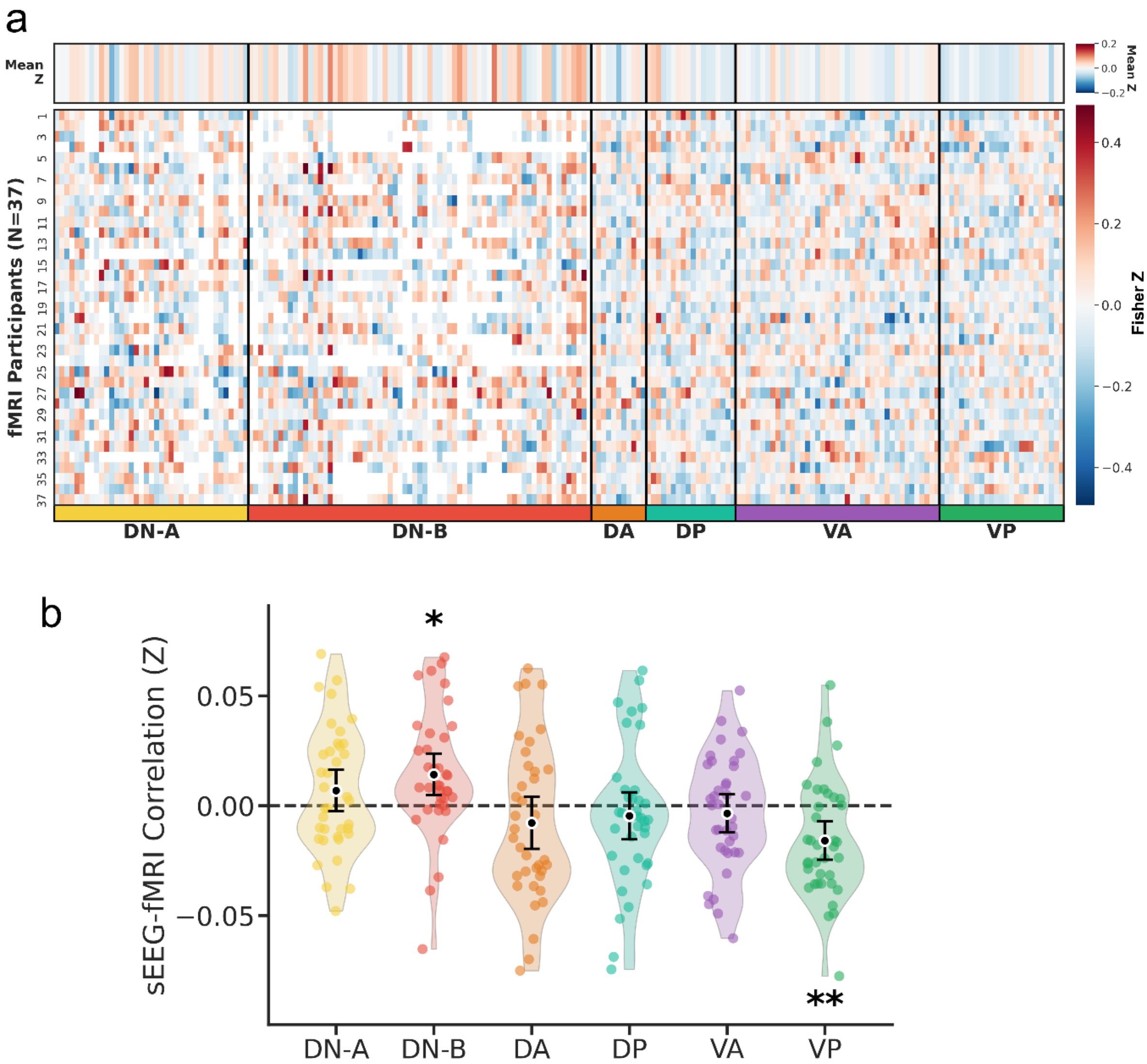

Figure S3. Cross-modal temporal correlation between sEEG and fMRI BOLD responses. (a) Brain-wide, electrode-level correlation matrix. The main heatmap displays the temporal correlation between the HRF-convolved sEEG HFA and the native-space fMRI BOLD signals across 37 independent fMRI participants (y-axis) and all valid sEEG recording sites (x-axis). Electrodes are grouped by cortical DN subsystems and thalamic ROIs. Color intensities reflect Fisher Z-transformed Pearson correlation coefficients. The top panel illustrates the mean Fisher Z across the fMRI cohort for each respective electrode. Notably, white spaces (NaNs) within the cortical DN regions denote electrodes where valid BOLD signals could not be extracted due to technical reasons (e.g., signal dropout at air-tissue interfaces or edge effects during nonlinear co-registration to individual native functional spaces). (b) Network-level average of the cross-modal temporal correlations. For each fMRI participant, Fisher Z values were first averaged across all valid electrodes within a given ROI to derive a subject-level mean. Each dot represents an individual fMRI participant. Error bars denote 95% confidence intervals. Statistical significance was evaluated across the fMRI cohort using one-sample t-tests against zero (two-tailed). * $p < 0.05$, ** $p < 0.01$ (FDR-corrected).

bioRxiv preprint doi: https://doi.org/10.64898/2026.07.28.741302; this version posted July 31, 2026. The copyright holder for this preprint (which was not certified by peer review) is the author/funder, who has granted bioRxiv a license to display the preprint in perpetuity. It is made available under a CC-BY 4.0 International license.

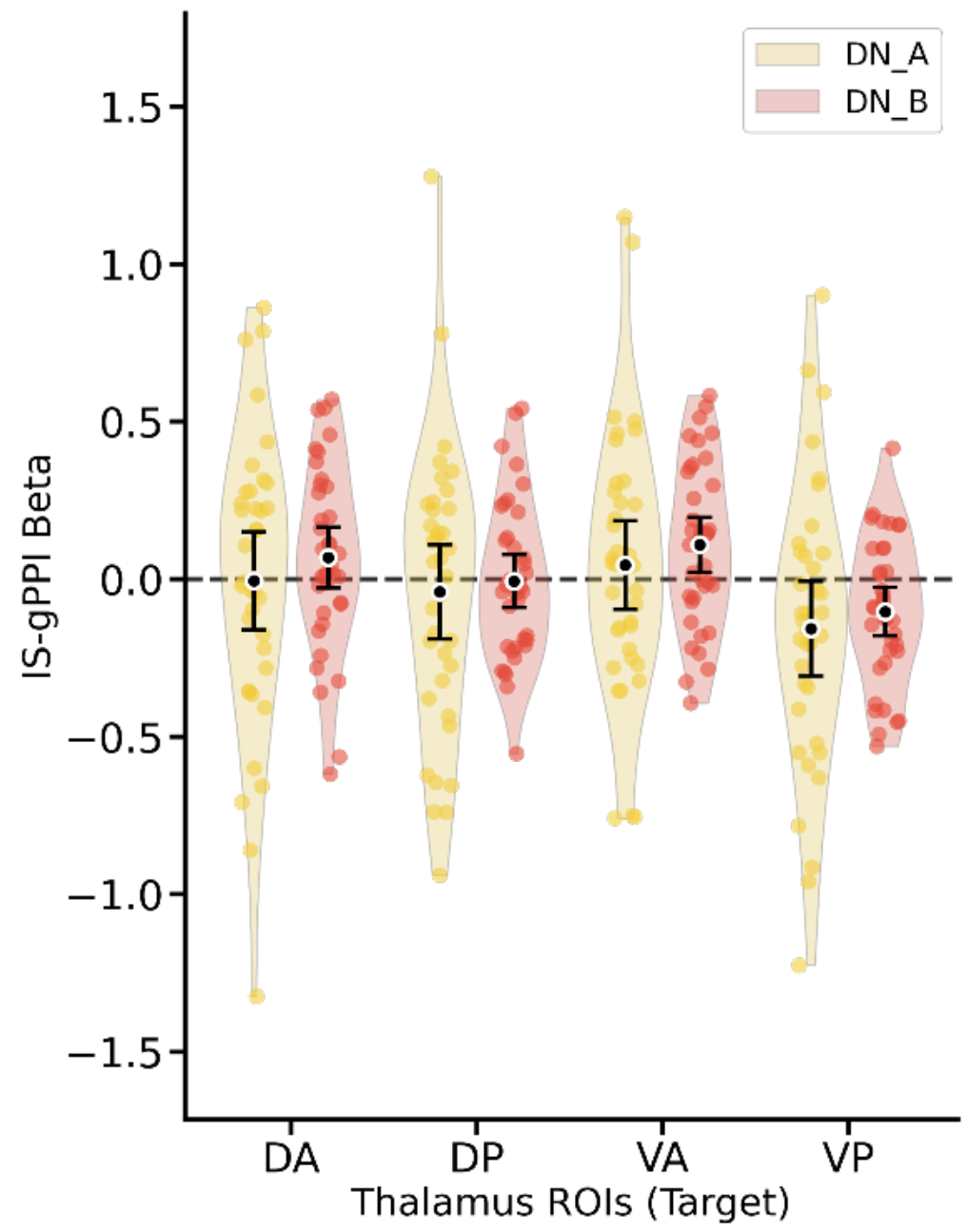

Figure S4. ROI-level fMRI IS-PPI results for corticothalamic (DN-to-Thalamus) connectivity during ToM. Violin plots display the task-modulated connectivity (IS-gPPI Beta) from default network subsystems to the four predefined thalamic ROIs. Error bars represent 95% confidence intervals, and each dot represents an individual subject.

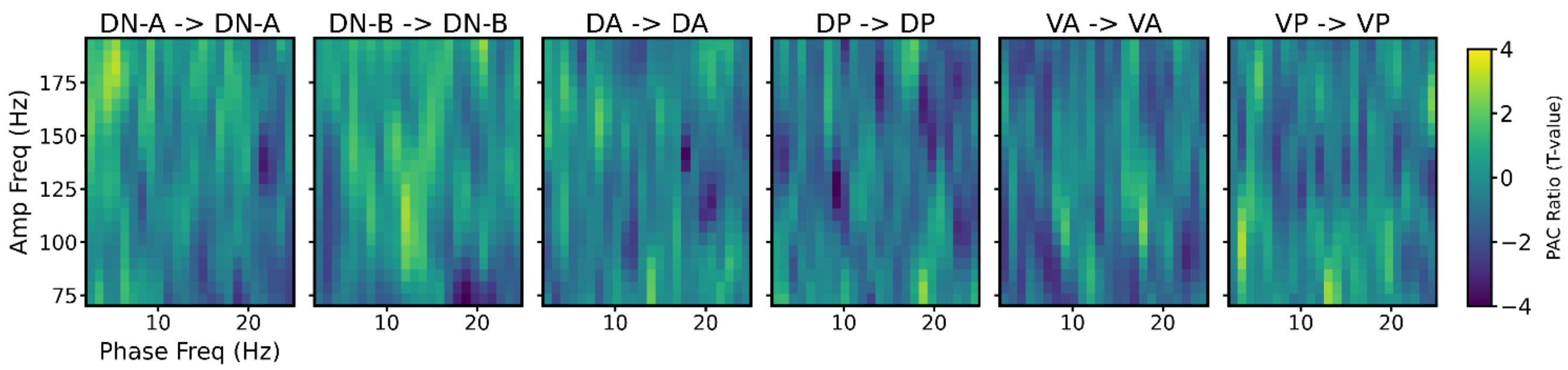

Figure S5. Local PAC within the DN and thalamic ROIs. ToM-related cross-frequency phase-amplitude coupling (PAC) from each ROI's phase to its own high-frequency amplitude. Color maps represent the group-level normalized PAC contrast ratio (comparing ToM events against non-ToM baseline periods, expressed as T-values).

bioRxiv preprint doi: https://doi.org/10.64898/2026.07.28.741302; this version posted July 31, 2026. The copyright holder for this preprint (which was not certified by peer review) is the author/funder, who has granted bioRxiv a license to display the preprint in perpetuity. It is made available under a CC-BY 4.0 International license.

Table S1 Timeline and descriptions of ToM events in the animated short film *Partly Cloudy* (Adapted from Richardson et al., 2018)

Event	Time	Duration (s)	Rephrased Description (Focusing on Mental States)
T1	4:00-4:10	10	Fleeing from the baby shark, Peck lands on a happier cloud. Gus observes them, inferring that they are mocking him together.
T2	2:46-3:02	16	Peck enviously watches another cloud create puppies. Gus misinterprets this as abandonment and looks anxious, causing Peck to feel embarrassed when caught staring.
T3	1:14-1:20	6	A crying baby experiences a shift in emotional state, becoming happy and comforted after receiving a helmet.
T4	4:42-4:56	14	Peck returns wearing football gear to resolve Gus's misunderstanding. He demonstrates that he didn't abandon Gus, but merely needed protective armor to safely transport Gus's hazardous creations.
T5	1:28-1:36	8	The scene shifts to Gus, visually depicting his feelings of loneliness and isolation compared to the other cheerful clouds.
T6	3:48-3:58	10	Startled by the dangerous baby shark Gus just created, Peck's attention is drawn to a neighboring cloud safely making chicks, prompting a contrast in expectations.
T7	1:50-1:54	4	Peck and Gus share a joyful reunion, mutually expressing happiness and reaffirming their bond of friendship.
